# A disproportionality analysis of adverse events caused by GnRHas from the FAERS and JADER databases

**DOI:** 10.3389/fphar.2024.1392914

**Published:** 2024-07-04

**Authors:** Shupeng Zou, Mengling Ouyang, Yazheng Zhao, Qian Cheng, Xuan Shi, Minghui Sun

**Affiliations:** Department of Pharmacy, Tongji Hospital, Tongji Medical College, Huazhong University of Science and Technology, Wuhan, Hubei, China

**Keywords:** GnRHas, FAERS, JADER, data mining, adverse events

## Abstract

**Background:**

Gonadotrophin-releasing hormone analogs (GnRHas) play a significant role in addressing gynecological diseases, central precocious puberty, and cancer. However, ensuring the safety of GnRHas in real-world applications requires continuous vigilance. In light of this, we undertook a disproportionality analysis focused on adverse events (AEs) associated with GnRHas using data from both the FDA Adverse Event Reporting System (FAERS) and the Japanese Adverse Drug Event Report (JADER). We evaluated GnRHas-associated AEs and characterized the clinical priority of unlisted AEs caused by each GnRHa from the different databases.

**Methods:**

In the disproportionality analysis, we applied two adjusted algorithms to identify signals related to GnRHas in the FAERS and JADER databases from 2004 to 2023. Additionally, we utilized the Statistical Analysis System (SAS, 9.4) to examine potential and high-aROR (adjusted reporting odds ratio) signals associated with GnRHas. We performed clinical priority assessment for suspicious PTs and an analysis of serious/non-serious outcomes. We also gathered information on the onset times of AEs linked with GnRHas from both databases.

**Results:**

From January 2004 to September 2023, FAERS and JADER recorded a total of 50,360,413 and 1,440,200 AEs, respectively. Employing two algorithms, the suspicious preferred terms (PTs) related to leuprolide (Leu) were 562 potential PTs (44 unlisted in specifications), followed by goserelin (Gos) with 189 PTs (28 unlisted), triptorelin (Tri) with 172 PTs (28 unlisted), and Leu-JADER with 85 PTs (10 unlisted). At the same PT level, the differences in GnRHas between the two databases were observed, such as cardiac failure, diabetes mellitus, liver disorder, dementia, suicidal ideation, interstitial lung disease, urinary disorders, and hypertensive crisis. In an analysis of serious vs. non-serious outcomes, a total of 43 AEs of Leu were more likely to be reported as serious AEs with *p* < 0.05 (such as asthenia, urinary retention, diabetes mellitus, interstitial lung disease, gait disturbance, and so on), following by Tri (6 AEs), and Gos (4 AEs). Based on the clinical priority score, 41 PTs of Leu, 26 PTs of Tri, 24 PTs of Gos, and 8 PTs of Leu-JADER were graded as weak. There were 3 PTs of Leu, 2 PTs of Tri, 4 PTs of Gos, and 2 PTs of Leu-JADER that were graded as moderate. Notably, in the assessment of the relevant evidence, 2 PTs (loss of libido and urinary tract toxicity caused by Leu), 1 PT (electrolyte imbalance caused by Tri), and 2 PTs (anorexia and suicidal ideation caused by Gos) showed a strong level of evidence with “++.” The differences in the signal strength of the same PTs from two databases were also worth noting. Moreover, the median onset time for GnRHas (Leu, Tri, and Gos) was 23 days (0, 298), 22 days (0, 181), and 217 days (29, 706), respectively, as median (Q1, Q3).

**Conclusion:**

An examination of two databases revealed suspicious AEs associated with GnRHas. Our study found potential new AE signals of GnRHas and supported continuous clinical monitoring, pharmacovigilance, regional differences, and further studies of GnRHas.

## Introduction

Hormone-dependent cancers, such as breast and prostate cancers, constitute over 20% of all cancers globally and over 35% of cancers in women (Sung et al., 2021; Rižner and Romano, 2023). Imbalances at any level of the hypothalamic–pituitary–gonadal (HPG) axis may lead to various hormone-dependent diseases (HDDs), including precocious puberty, infertility, hormone-dependent cancers, polycystic ovarian syndrome, endometriosis, and uterine fibroids (Newton et al., 2018). Gonadotropin-releasing hormone analogs (GnRHas), synthetic derivatives of the hormone gonadorelin, stimulate the pituitary gland within the brain, promoting the production of reproductive hormones, including luteinizing hormone (LH) and follicle-stimulating hormone (FSH) (Veth et al., 2023).

Hormone-dependent diseases also include noncancerous conditions such as precocious puberty, endometriosis, uterine fibroids, Cushing’s syndrome, and polycystic ovarian syndrome (PCOS) (Zondervan et al., 2020; Rižner and Romano, 2023). Although these conditions are non-life-threatening, they significantly impact quality of life. Hormonal therapy refers to both androgen deprivation therapy for prostate cancer and endocrine therapy for breast cancer (Okwuosa et al., 2021). Approximately 10% of women of reproductive age are affected by endometriosis. Additionally, central precocious puberty (CPP) is a prevalent pediatric hormone-dependent disorder (HDD) that can result in the premature activation of the hypothalamic–pituitary–gonadal (HPG) axis. It is noteworthy that approximately 90% of girls with CPP experience idiopathic central precocious puberty (ICPP) (Zondervan et al., 2020; Hou et al., 2024). CPP could affect the physical and psychological health of children. Since the 1980s, GnRHas, acting as inhibitors binding to the GnRH receptor in the anterior pituitary, have been extensively employed for the treatment of CPP (Carel et al., 2009). Furthermore, the combination of GnRHas with chemotherapy in premenopausal patients with breast cancer has been shown to decrease the risk of premature ovarian insufficiency, which also contributes to the restoration of ovarian function (Zong et al., 2022). However, the prolonged administration of GnRHas, including leuprorelin, triptorelin, goserelin, buserelin, and histrelin, leads to the suppression of gonadal function. Consequently, this influence extends to hormone levels within the HPG axis, including testosterone, estrogen, and progesterone (Harris et al., 2022; Raja et al., 2022). GnRHas also could result in a reduction in ovarian hyperstimulation syndrome and bone mineral density, like menopausal symptoms, since they momentarily halt the synthesis of reproductive hormones (Fatemi and Garcia-Velasco, 2015; Veth et al., 2023). A study examining bone mineral density (BMD) in short children undergoing growth hormone (GH) and GnRHas treatment revealed that 2 years of GnRHas supplementation, alongside GH treatment, had no adverse effects on BMD or body composition (Lem et al., 2013). In addition to hormone-related symptoms such as hot flushes, osteopenia, and reproductive system disorders, what other adverse events (AEs) could potentially be induced by GnRHas?

In a Cochrane meta-analysis, aside from the risk of hot flushes (RR 1.62; 95% CI 0.87–3.02), the use of GnRHas may also be associated with higher incidence of vasodilatation (RR 2.69; 95% CI 1.51–4.81), headache (RR 3.55; 95% CI 1.09–11.53), and sleep disturbances (RR 2.31; 95% CI 1.33–4.02) compared to placebo (Veth et al., 2023). In a phase-2 randomized clinical trial (RCT), the combination with GnRH analogs was found to significantly elevate the risk of erectile dysfunction (30.8%), decreased libido (42.3%), bone pain (23.1%), myalgia (17.3%), and sleeping disorders (7.7%) (Reinisch et al., 2021).

Recently, two large real-world spontaneous adverse event reporting databases—the US Food and Drug Administration (FDA) Adverse Event Reporting System (FAERS) and the Japanese Adverse Drug Event Report (JADER) —have collected abundant AEs reports from different cohorts (one mainly from America and one mainly from the Japan) (Neishi et al., 2023). These two databases are reported to have different features, such as FAERS having many non-serious AEs reported from non-health care professionals while JADER has many serious AEs many serious AEs reported by medical personnel (Nomura et al., 2015; Nagai and Ishikawa, 2021). The differences of reporting areas and reporters may result in different tendencies of these databases.

## Methods

### Data sources

We collected the data of all GnRHas (leuprorelin, triptorelin, goserelin, buserelin, and histrelin) from FAERS and JADER from January 2004 to September 2023. Medical personnel, consumers, manufacturers, and others submit AEs and updates to these databases as spontaneous report systems (Zou et al., 2023b). From JADER, this study defined AEs using the PTs from the standardized Medical Dictionary for Regulatory Activities/Japanese version (MedDRA/J 26.0). The data files of FAERS and JADER databases are available from their official websites (https://fis.fda.gov/extensions/FPD-QDE-FAERS/FPD-QDE-FAERS.html; https://www.info.pmda.go.jp/fukusayoudb/CsvDownload).

### Data extraction and descriptive analysis

We searched the disproportionality signals of GnRHas and their types in medical subject headings [MeSH] in Supplementary Table S1 from FAERS and JADER. We removed duplicate reports sharing the same identifier number from the demographic file and conducted an interlinkage of the reaction file with MedDRA using the PT code (Zou et al., 2023b). All data were imported into SAS software (v9.4), with the primary id as the primary link field (primary key) between different data files—patient demographic file [DEMO], drug file [DRUG], adverse events file [REAC], outcome file [OUTC], report source file [RPSR], drug therapy file [THER], and drug indication [INDI] (Shu et al., 2023a).

We screened cases by generic names and trade names (searching by MeSH in Supplementary Table S1) in the DRUG file and chose role_cod as the primary suspected (PS) in FAERS. The process of data mining is shown in Supplementary Figure S1. Because the quantity of some GnRHas (buserelin and histrelin) was too small, we ultimately only analyzed other GnRHas (leuprorelin, triptorelin, and goserelin). It is noteworthy that the serious outcomes included death, threats to life, hospitalization, disability, and other serious outcomes (Shu et al., 2022). We thus performed a statistical analysis of serious vs. non-serious (Burk et al., 2023).

### Clinical prioritization of signals

According to the designated medical event (DME) and important medical event (IME) lists from the European Medicines Agency (EMA), we created a semiquantitative score method to rank the significant disproportionality PTs in order to display special AEs (Shu et al., 2023b). The significant disproportionate AEs with weak, moderate, or strong clinical priority depended on scores of 0 and 3, 4 and 6, or 7 and 9, respectively (Gatti et al., 2021; Shu et al., 2022).

### Statistical analysis

The adjusted reporting odds ratio (aROR) and information component (IC) of Bayesian confidence propagation neural network (BCPNN), two of the algorithms used in the disproportionality analysis, were based on the 2 × 2 table calculation principle in Supplementary Table S1 (Nagai and Ishikawa, 2021; Zou et al., 2023a; Jain et al., 2023). For the sake of robustness, statistical shrinkage transformation was performed, with the calculation formulas for aROR and IC after transformation displayed in Supplementary Table S1 (Noren et al., 2013; Chen et al., 2021). We used the following statistical methods to observe the value of *P*: Pearson’s chi-squared (χ^2^) test, Fisher’s exact test, and Mann–Whitney U test (Sahai and Khurshid, 1995; Chau et al., 2017; Shi et al., 2023). The time-to-onset of adverse events used the formula Time-to-onset = Event onset date (EVENT_DT) –Therapy start date (START_DT), while we deleted missing or unreasonable data like the negative number of time-to-onset (24).

## Results

### Descriptive analysis

There were 50,360,413 AEs and 1,440,200 AEs reported in the FAERS and JADER databases from January 2004 to September 2023. After data mining and calculation in Supplementary Figure S1, 191,287 AEs of GnRHas were collected in our study, including leuprorelin (167,046), triptorelin (8,947), goserelin (12,042), leuprorelin (3,252 AEs from JADER), and goserelin (784 AEs from JADER). The characteristics of AEs, including age, sex, outcomes, indications, and the reports’ countries, are shown in Table 1. In terms of gender distribution, the proportions of male individuals were higher than female individuals for other GnRH analogs (Leu, Gos, Leu-JADER, and Gos-JADER) with percentages of 60.67% vs. 32.05%, 42.80% vs. 19.99%, 79.18% vs. 20.30%, and 76.40% vs. 23.34%, respectively. The reason for this phenomenon may be that those GnRHas (Leu, Gos, Leu-JADER, and Gos-JADER) were used more for prostate cancer. Regarding serious reports of each GnRHas (Leu, Tri, and Gos from FAERS), the serious proportion (52.59%, 56.86%, and 89.23%) was larger than the non-serious (47.41%, 43.14%, and 10.77%), especially Gos. Finally, suspicious PTs conforming to the two algorithms (N ≥ 3, aROR_025_ > 1 and IC_025_ > 0) are displayed in Supplementary Table S2, following by Leu (562 PTs and 44 in new), Gos (189 PTs and 28 in new), Tri (172 PTs and 28 in new), and Leu-JADER (85 PTs and 10 in new).

**TABLE 1:** Characteristics of GnRHas from FAERS and JADER databases (2004 to September 2023).

Characteristics, number (%)	FAERS			JADER	
	Leuprorelin	Triptorelin	Goserelin	Leuprorelin	Goserelin
Number of events	167,046	8,947	12,042	3,252	784
Gender					
Female	53,537 (32.05)	4,471 (49.97)	2,394 (19.99)	660 (20.30)	183 (23.34)
Male	101,343 (60.67)	3,799 (42.46)	5,154 (42.80)	2,575 (79.18)	599 (76.40)
Not specified	12,166 (7.28)	677 (7.57)	4,494 (37.32)	17 (0.52)	2 (0.26)
Age (years)					
<18	4,728 (2.83)	1,862 (20.81)	30 (0.25)	73 (2.24)	0
18≤ and <45	24,381 (14.60)	684 (7.65)	999 (8.30)	470 (14.45)	146 (18.62)
≥45 and <65	14,288 (8.55)	1,011 (11.30)	1,180 (9.80)	448 (13.78)	77 (9.82)
≥65	52,895 (31.66)	3,524 (39.39)	3,187 (26.47)	1,879 (57.78)	456 (58.16)
Not specified	70,754 (42.36)	1,866 (20.86)	6,646 (55.19)	382 (11.75)	105 (13.39)
Serious reports					
Serious	87,878 (52.61)	5,087 (56.86)	10,745 (89.23)	NA	NA
Non-serious	79,168 (47.39)	3,860 (43.14)	1,297 (10.77)	NA	NA
Outcome					
Hospitalization	25,556 (15.30)	1,867 (20.87)	2,477 (20.57)	NA	NA
Death	6,876 (4.12)	187 (2.09)	1,316 (10.93)	86 (2.64)	60 (7.65)
Life-threatening	750 (0.45)	44 (0.49)	133 (1.10)	NA	NA
Disability	5,228 (3.13)	277 (3.10)	1,068 (8.87)	NA	NA
RIPPI	585 (0.35)	3 (0.03)	166 (1.38)	NA	NA
Other serious events	36,491 (21.84)	2,327 (26.01)	4,300 (35.71)	NA	NA
Indications					
Unknown indication	54,143 (32.41)	2,965 (33.14)	1,942 (16.13)	NA	NA
Prostate cancer	38,990 (23.34)	1,559 (17.42)	3,182 (26.42)	1,580 (48.59)	379 (48.34)
Endometriosis	7,759 (4.64)	44 (0.49)	193 (1.60)	67 (2.06)	28 (3.57)
Uterine leiomyoma	2,590 (1.55)	11 (0.12)	68 (0.56)	193 (5.93)	15 (1.91)
Precocious puberty	1,680 (1.01)	215 (2.40)	4 (0.03)	29 (0.89)	0
Breast cancer	719 (0.43)	168 (1.88)	787 (6.54)	121 (3.72)	49 (6.25)
Reported person					
Health professional					
Physician	25,280 (15.13)	1,588 (17.75)	3,478 (28.88)	2,727 (83.86)	652 (83.16)
Pharmacist	30,262 (18.12)	1,400 (15.65)	1,318 (10.95)	337 (10.36)	52 (6.63)
Other professional	11,093 (6.64)	1,007 (11.26)	1,191 (9.89)	112 (3.44)	24 (3.06)
Non-professional					
Consumer	94,463 (56.55)	4,661 (52.10)	4,287 (35.60)	69 (2.12)	23 (2.93)
Unknown	5,948 (3.56)	291 (3.25)	1,759 (14.60)	7 (0.22)	33 (4.21)
Reporting year					
2023 Q1–Q3	23,017 (13.77)	1,204 (15.54)	855 (7.10)	33 (1.01)	8 (1.02)
2022	19,987 (11.96)	1,243 (18.36)	858 (7.13)	167 (5.14)	58 (7.40)
2021	16,951 (10.15)	1,286 (19.16)	963 (8.00)	228 (7.01)	37 (4.72)
2020	15,127 (9.06)	1,054 (10.78)	693 (5.75)	251 (7.72)	28 (3.57)
2019	10,345 (6.19)	1,752 (15.64)	721 (5.99)	250 (7.69)	41 (5.23)
2018	8,761 (5.24)	558 (7.70)	1,078 (8.95)	262 (8.06)	43 (5.48)
2017	9,312 (5.57)	335 (1.67)	672 (5.58)	279 (8.58)	53 (6.76)
2016	11,082 (6.63)	214 (2.39)	723 (6.00)	296 (9.10)	39 (4.97)
2015	12,130 (7.26)	156 (1.74)	816 (6.78)	134 (4.12)	30 (3.83)
2014	10,620 (6.36)	54 (0.60)	707 (5.87)	128 (3.94)	48 (6.12)
2013	6,374 (3.82)	15 (0.17)	529 (4.39)	75 (2.31)	64 (8.16)
2012	5,075 (3.04)	84 (0.94)	445 (3.70)	89 (2.74)	22 (2.81)
2011	4,988 (2.99)	82 (0.92)	433 (3.60)	50 (1.54)	20 (2.55)
2010	4,614 (2.76)	81 (0.91)	270 (2.24)	111 (3.41)	34 (4.34)
2009	3,163 (1.89)	117 (1.31)	378 (3.14)	126 (3.87)	34 (4.34)
2008	1,752 (1.05)	189 (2.11)	465 (3.86)	171 (5.26)	44 (5.61)
2007	1,124 (0.67)	161 (1.80)	504 (4.19)	140 (4.31)	34 (4.34)
2006	837 (0.50)	118 (1.32)	334 (2.77)	143 (4.40)	37 (4.72)
2005	663 (0.40)	134 (1.50)	283 (2.35)	176 (5.41)	44 (5.61)
2004	1,124 (0.67)	110 (1.23)	315 (2.62)	143 (4.40)	66 (8.42)

GnRHas, gonadotropin-releasing hormone analogues; FAERS, FDA, adverse event reporting system; JADER, Japanese adverse drug event report; RIPPI, required intervention to prevent permanent impairment/damage; Q, quarter; NA, Not Applicable (for relevant criterias).

### Signals of system organ class

**FIGURE 1:**
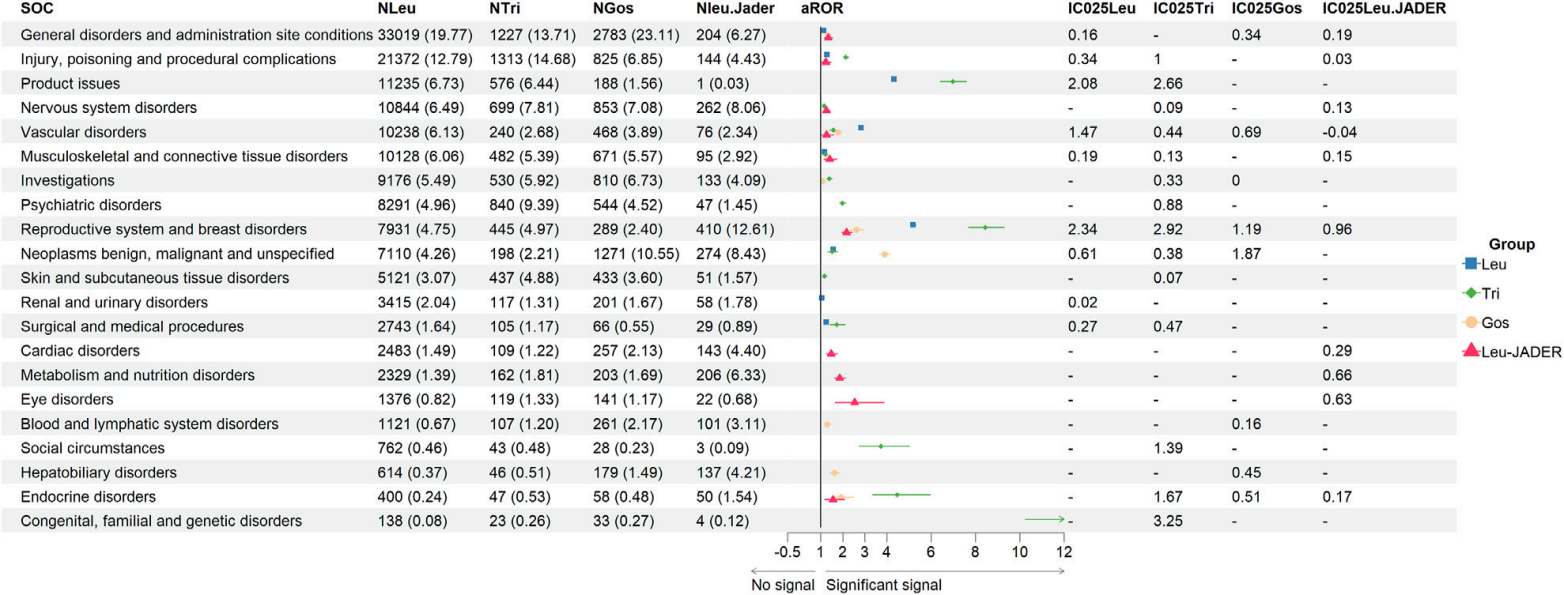
Signal strength of adverse events of GnRHas at the SOC level from FAERS database. -, showed a negative signal (aROR_025_ ≤ 1 and IC_025_ ≤ 0); GnRHas, gonadotropin-releasing hormone analogs; SOC, system organ class; FAERS, FDA Adverse Event Reporting System; N, number of target adverse events; Leu, leuprolide; Tir, triptorelin; Gos, goserelin; aROR, adjusted reporting odds ratio; IC_025_, adjusted lower limit of 95% confidence interval of the information component of BCPNN; BCPNN, Bayesian confidence propagation neural network.

The system organ class (SOC) of all GnRHas, which conformed to the criterion (aROR_025_ > 1 or IC_025_ > 0), is showed in Figure 1. We detected that GnRHas-induced AEs occurred in 21 targeted organ systems, including Leu (9 SOCs), Tri (15 SOCs), Gos (8 SOCs), and Leu-JADER (11 SOCs). From Figure 1, we found that the SOC signals of Tri were the largest, including reproductive system disorders (aROR 8.44, 95% CI 7.68–9.29), product issues (aROR 6.97, 95% CI 6.41–7.59), endocrine disorders (aROR 4.46, 95% CI 3.35–5.94), social circumstances (aROR 3.72, 95% CI 2.76–5.02), injury conditions (aROR 2.14, 95% CI 2.02–2.27), psychiatric disorders (aROR 1.99, 95% CI 1.85–2.14), and skin disorders (aROR 1.17, 95% CI 1.06–1.29). Comparing FAERS and JADER, the SOC signals of Leu (FAERS) were more prominent in reproductive system disorders (aROR 5.28, 95% CI 5.07–5.30), product issues (aROR 4.32, 95% CI 4.24–4.41), vascular disorders (aROR 2.83, 95% CI 2.77–2.89), and renal/urinary disorders (aROR 1.05, 95% CI 1.02–1.09). Some SOC signals of Leu (JADER) demonstrated stronger signal values, including eye (aROR 2.53, 95% CI 1.66–3.85), metabolic (aROR 1.86, 95% CI 1.61–2.14), and cardiac disorders (aROR 1.48, 95% CI 1.25–1.76).

### Signals of same preferred terms from FAERS

**FIGURE 2:**
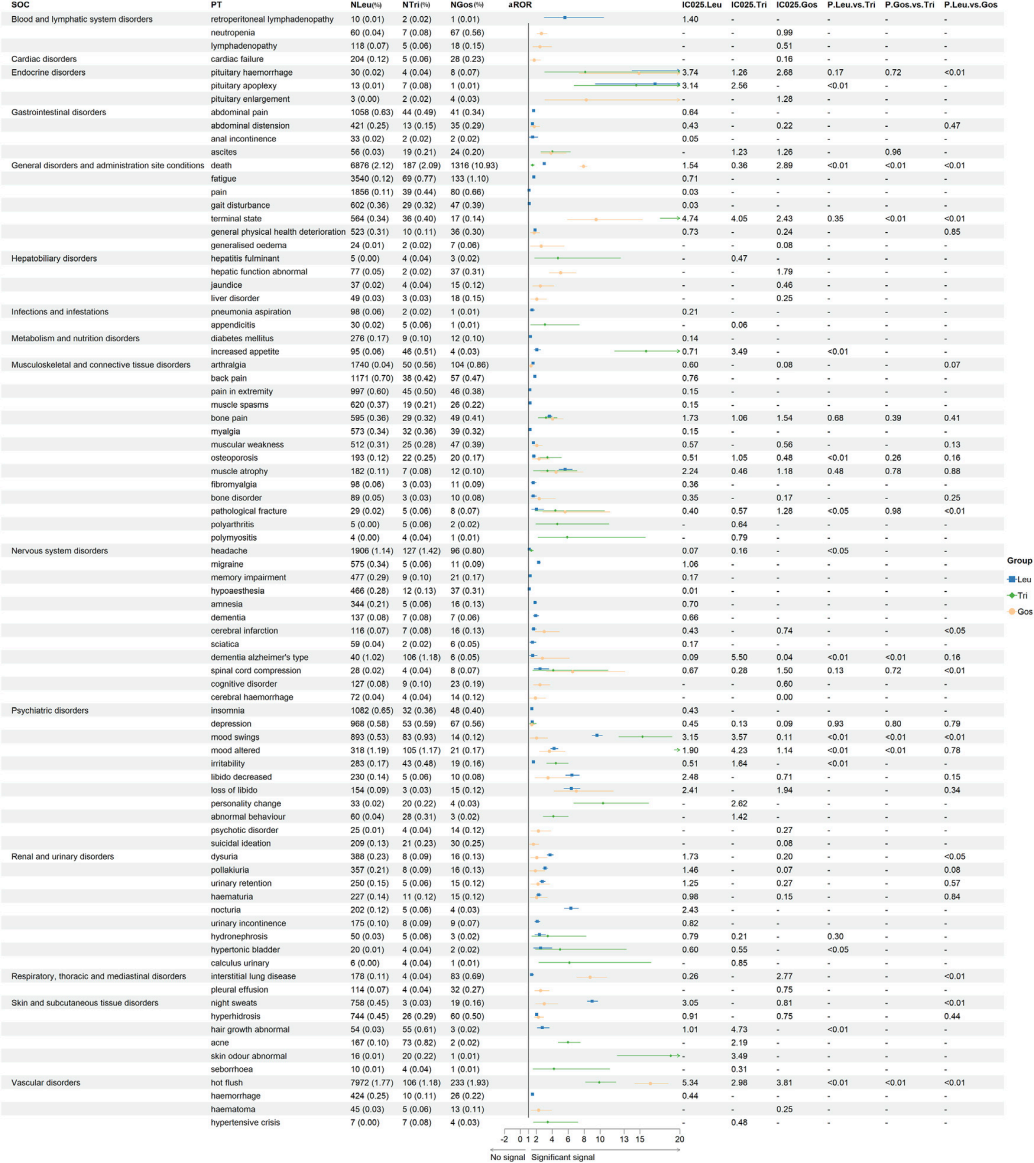
Signal strength of the same adverse events of GnRHas at the PT level from FAERS database. -, showed a negative signal (aROR_025_ ≤ 1 or IC_025_ ≤ 0); *p* < 0.05, showed the significant difference by Pearson χ^2^ test. GnRHas, gonadotropin-releasing hormone analogs; SOC, system organ class; PT, preferred term; FAERS, FDA Adverse Event Reporting System; N, cases of target adverse events; Leu, leuprolide; Tir, triptorelin; Gos, goserelin; aROR, adjusted reporting odds ratio; IC_025_, adjusted lower limit of 95% confidence interval of the information component of BCPNN; BCPNN, Bayesian confidence propagation neural network.

Figure 2 showed the same PTs (84 PTs from 15 SOCs) of GnRHas (Leu, Tri, and Gos) from FAERS, comparing the statistical significance of their signal strength by Pearson χ^2^ test. To facilitate analysis of this data, the negative signals were marked “-”. In blood system disorders, Leu and Gos showed stronger PTs signals, including retroperitoneal lymphadenopathy, neutropenia, and lymphadenopathy. In cardiac disorders, suspected PT of cardiac failure (Gos) detected the stronger signal (aROR 1.73, 95% CI 1.20–2.51). In the PT of death, the order of signal from strong to weak is Gos (aROR 7.90, 95% CI 7.46–8.37), Leu (aROR 2.98, 95% CI 2.91–3.06), and Tri (aROR 1.51, 95% CI 1.31–1.75). In hepatobiliary disorders, Leu and Gos showed stronger PTs signals than hepatitis fulminant, jaundice, and liver disorder. In musculoskeletal and connective tissue disorders, partial PTs only showed Leu having positive signals as back pain, extreme pain, muscle spasms, myalgia, and fibromyalgia. Moreover, two PTs (polyarthritis and polymyositis) were observed to be significantly associated with Tri.

In metabolic and nutrition disorders, only Leu showed a positive signal for diabetes mellitus (aROR 1.26, 95% CI 1.12–1.42). In the nervous system and psychiatric disorders, seven PTs showed a unique and positive signal for Leu involving migraine, memory impairment, hypoaesthesia, amnesia, dementia, sciatica, and insomnia. In renal and urinary disorders, the dysuria signal of Leu was larger than that of Gos (aROR 3.72 vs. 2.06, *p* < 0.05). In interstitial lung disease and pleural effusion, Gos (aROR 8.75 and 2.54) showed the largest signals. In skin disorders, Tri detected the largest signals, including abnormal hair growth, acne, abnormal skin odor, and seborrhea. Moreover, hot flush of different GnRHas also showed different aROR values, and Leu had the highest value of IC_025_ (5.34), followed by Gos (IC_025_, 3.81) and Tri (IC_025_, 2.98).

### Serious vs. non-serious cases from FAERS

As shown in Table 2, we performed an analysis of serious vs. non-serious cases of each GnRHa (leuprorelin, triptorelin, and goserelin from FAERS) with the same PTs. There were statistically significant differences in gender, age (69 vs. 61 years; *p* < 0.001, 63 vs. 9 years; *p* < 0.001; 70 vs. 59 years; *p* < 0.001) and body weight (79 vs. 73 kg; *p* < 0.001, 70 vs. 78 years; *p* = 0.003; 63 vs. 75 years; *p* < 0.001) between severe and non-severe cases of GnRHas-associated AEs from FAERS. A total of 43 AEs of Leu were more likely to be reported as serious AEs with *p* < 0.05 (such as asthenia, urinary retention, diabetes mellitus, interstitial lung disease, gait disturbance, and so on), following by Tri (6 AEs), and Gos (4 AEs). Back pain was reported as non-serious AEs with *p* > 0.05, as Leu (χ^2^ = 0.013, *p* = 0.91), Tri (χ^2^ = 2.080, *p* = 0.15), and Gos (χ^2^ = 0.635, *p* = 0.43). For Lue, 16 AEs showed non-serious events with *p* > 0.05, including back pain, bone pain, memory impairment, muscle atrophy, musculoskeletal pain, lower abdominal pain, abscess, groin pain, osteopenia, micturition urgency, sciatica, apathy, decreased proctalgia, exercise tolerance, increased fat tissue, and aggression. Like Leu, it was noteworthy that all outcomes for AEs of cerebral infarction (*N* = 116), urinary tract obstruction (*N* = 69), marasmus (*N* = 57), urinary bladder hemorrhage (*N* = 53), hydronephrosis (*N* = 50), aortic aneurysm (*N* = 38), and pituitary hemorrhage (*N* = 30) were severe cases.

**TABLE 2:** Differences in clinical characteristics of serious and non-serious reports of each GnRHa (*n* ≥ 30).

	Leuprorelin				Triptorelin				Goserelin			
	Serious(*n* = 87,878)	Non-serious(*n* = 79,199)	Statistic	*p*	Serious(*n* = 5,087)	Non-serious(*n* = 3,860)	Statistic	*p*	Serious(*n* = 10,745)	Non-serious(*n* = 1,297)	Statistic	*p*
Gender, *n*	—	—	—	—	—	—	—	—	—	—	—	—
Female	24,396	29,141	1561.062^a^	<0.001^b^	1,988	2,483	559.540^a^	<0.001^b^	2,034	360	56.607^a^	<0.001^b^
Male	58,590	42,722	2827.972^a^	<0.001^b^	2,822	977	817.321^a^	<0.001^b^	4,778	366	124.863^a^	<0.001^b^
Age, years (median, IQR)	69 (43–104)	61 (32–106)	−51.476^c^	<0.001^d^	63 (33–96)	9 (8–92)	−36.257^c^	<0.001^d^	70 (46–105)	59 (43–96)	−7.567^c^	<0.001^d^
Weight, kg (median, IQR)	79 (66–325)	73 (61–270)	−27.579^c^	<0.001^d^	70 (45–246)	78 (55–195)	−2.982^c^	0.003^d^	63 (36–295)	75 (62–168)	−7.567^c^	<0.001^d^
Types of same AEs, *n* ≥ 30	—	—	—	—	—	—	—	—	—	—	—	—
Hot flush	2,081	5,891	2356.733^a^	<0.001^b^	42	64	12.989^a^	<0.001^b^	184	49	26.022^a^	<0.001^b^
Fatigue	1,512	2,028	141.752^a^	<0.001^b^	35	34	1.066^a^	0.302^b^	112	21	3.525^a^	0.060^b^
Prostatic-specific antigen increased	1,223	564	181.809^a^	<0.001^b^	27	10	3.934^a^	0.047^b^	35	16	22.619^a^	<0.001^b^
Asthenia	1,052	627	68.831^a^	<0.001^b^	57	11	20.313^a^	<0.001^b^	99	7	1.932^a^	0.165^b^
Arthralgia	792	948	35.351^a^	<0.001^b^	41	9	12.958^a^	<0.001^b^	89	15	1.456^a^	0.228^b^
Back pain	614	557	0.013^a^	0.91^b^	26	12	2.080^a^	0.149^b^	49	8	0.635^a^	0.426^b^
Weight increased	549	992	179.672^a^	<0.001^b^	34	115	71.568^a^	<0.001^b^	55	13	4.958^a^	0.026^b^
Headache	535	1,371	465.227^a^	<0.001^b^	52	75	13.298^a^	<0.001^b^	82	14	1.464^a^	0.226^b^
General health deterioration	496	27	375.447^a^	<0.001^b^	NA	NA	NA	NA	36	0	NA	0.029^e^
Pain in extremity	482	515	7.274^a^	0.007^b^	28	17	0.531^a^	0.466^b^	42	4	0.047^f^	0.829^g^
Depression	475	493	4.858^a^	0.028^b^	45	8	17.100^a^	<0.001^b^	57	10	1.210^a^	0.271^b^
Abdominal pain	450	608	43.255^a^	<0.001^b^	28	16	0.828^a^	0.363^b^	36	5	0.087^a^	0.768^b^
Insomnia	421	661	81.838^a^	<0.001^b^	23	9	2.953^a^	0.086^b^	42	6	0.150^a^	0.699^b^
Gait disturbance	380	222	26.847^a^	<0.001^b^	NA	NA	NA	NA	44	3	0.542^f^	0.461^g^
Bone pain	324	271	0.825^a^	0.364^b^	NA	NA	NA	NA	41	8	1.580^a^	0.209^b^
Muscular weakness	323	189	22.661^a^	<0.001^b^	NA	NA	NA	NA	43	4	0.070^f^	0.791^g^
Hyperhidrosis	311	433	34.937^a^	<0.001^b^	NA	NA	NA	NA	53	7	0.050^a^	0.822^b^
Mobility decreased	297	75	110.969^a^	<0.001^b^	NA	NA	NA	NA	NA	NA	NA	NA
Myalgia	272	301	6.064^a^	0.014^b^	23	9	2.953^a^	0.086^b^	33	6	0.867^a^	0.352^b^
Hypoaesthesia	271	195	5.788^a^	0.016^b^	NA	NA	NA	NA	36	0	NA	0.029^e^
Diabetes mellitus	259	17	188.611^a^	<0.001^b^	NA	NA	NA	NA	NA	NA	NA	NA
Urinary retention	246	4	208.836^f^	<0.001^g^	NA	NA	NA	NA	NA	NA	NA	NA
Mood swings	241	652	236.167^a^	<0.001^b^	22	61	31.461^a^	<0.001^b^	NA	NA	NA	NA
Memory impairment	240	237	1.000^a^	0.317^b^	NA	NA	NA	NA	NA	NA	NA	NA
Hemorrhage	239	158	9.229^a^	0.002^b^	NA	NA	NA	NA	NA	NA	NA	NA
Night sweats	232	526	147.688^a^	<0.001^b^	NA	NA	NA	NA	NA	NA	NA	NA
Amnesia	230	114	28.127^a^	<0.001^b^	NA	NA	NA	NA	NA	NA	NA	NA
Migraine	227	348	39.829^a^	<0.001^b^	NA	NA	NA	NA	NA	NA	NA	NA
Dysuria	224	164	4.112^a^	0.043^b^	NA	NA	NA	NA	NA	NA	NA	NA
Hematuria	202	25	120.726^a^	<0.001^b^	NA	NA	NA	NA	NA	NA	NA	NA
Muscle spasms	196	424	109.909^a^	<0.001^b^	NA	NA	NA	NA	NA	NA	NA	NA
Suicidal ideation	183	26	102.596^a^	<0.001^b^	NA	NA	NA	NA	28	2	0.186^f^	0.666^g^
Disease progression	182	3	153.856^f^	<0.001^g^	32	0	NA	<0.001^e^	49	2	1.835^f^	0.175^g^
Interstitial lung disease	177	1	154.932^f^	<0.001^g^	NA	NA	NA	NA	82	1	6.987^f^	0.008^g^
Abdominal distension	165	256	30.419^a^	<0.001^b^	NA	NA	NA	NA	30	5	0.451^a^	0.502^b^
Pollakiuria	164	193	6.363^a^	0.012^b^	NA	NA	NA	NA	NA	NA	NA	NA
Osteoporosis	154	39	57.317^a^	<0.001^b^	NA	NA	NA	NA	NA	NA	NA	NA
Dementia	130	7	98.367^a^	<0.001^b^	NA	NA	NA	NA	NA	NA	NA	NA
Urinary incontinence	118	57	15.456^a^	<0.001^b^	NA	NA	NA	NA	NA	NA	NA	NA
Cerebral infarction	116	0	—	<0.001^e^	NA	NA	NA	NA	NA	NA	NA	NA
Adverse drug reaction	115	231	52.124^a^	<0.001^b^	NA	NA	NA	NA	NA	NA	NA	NA
Pleural effusion	113	1	97.183^f^	<0.001^g^	NA	NA	NA	NA	NA	NA	NA	NA
Depressed mood	111	133	4.948^a^	0.026^b^	NA	NA	NA	NA	NA	NA	NA	NA
Muscle atrophy	103	79	1.167^a^	0.28^b^	NA	NA	NA	NA	NA	NA	NA	NA
Musculoskeletal pain	103	89	0.085^a^	0.771^b^	NA	NA	NA	NA	NA	NA	NA	NA
Gait inability	95	11	58.319^a^	<0.001^b^	NA	NA	NA	NA	NA	NA	NA	NA
Sleep disorder	90	126	10.364^a^	0.001^b^	NA	NA	NA	NA	NA	NA	NA	NA
Hypophagia	85	8	56.187^a^	<0.001^b^	NA	NA	NA	NA	NA	NA	NA	NA
Nocturia	84	118	9.839^a^	0.002^b^	NA	NA	NA	NA	NA	NA	NA	NA
Mood altered	81	237	94.026^a^	<0.001^b^	21	84	58.839^a^	<0.001^b^	NA	NA	NA	NA
Emotional disorder	80	158	34.451^a^	<0.001^b^	8	46	39.147^a^	<0.001^b^	NA	NA	NA	NA
Irritability	79	204	69.266^a^	<0.001^b^	13	30	12.486^a^	<0.001^b^	NA	NA	NA	NA
Hepatic function abnormal	74	3	56.751^f^	<0.001^g^	NA	NA	NA	NA	37	0	NA	0.029^e^
Urinary tract obstruction	69	0	—	<0.001^e^	NA	NA	NA	NA	NA	NA	NA	NA
Libido decreased	68	162	49.003^a^	<0.001^b^	NA	NA	NA	NA	NA	NA	NA	NA
Anger	67	88	5.465^a^	0.019^b^	NA	NA	NA	NA	NA	NA	NA	NA
Loss of libido	67	87	5.109^a^	0.024^b^	NA	NA	NA	NA	NA	NA	NA	NA
Fibromyalgia	66	32	8.556^a^	0.003^b^	NA	NA	NA	NA	NA	NA	NA	NA
Abdominal pain lower	63	58	0.014^a^	0.907^b^	NA	NA	NA	NA	NA	NA	NA	NA
Bone disorder	62	27	10.402^a^	0.001^b^	NA	NA	NA	NA	NA	NA	NA	NA
Incontinence	61	22	14.544^a^	<0.001^b^	NA	NA	NA	NA	NA	NA	NA	NA
Marasmus	57	0	—	<0.001^e^	NA	NA	NA	NA	NA	NA	NA	NA
Pulmonary mass	57	3	41.599^f^	<0.001^g^	NA	NA	NA	NA	NA	NA	NA	NA
Feeling cold	56	83	8.455^a^	0.004^b^	NA	NA	NA	NA	NA	NA	NA	NA
Inguinal hernia	54	7	31.592^a^	<0.001^b^	NA	NA	NA	NA	NA	NA	NA	NA
Cyst	54	22	10.387^a^	0.001^b^	NA	NA	NA	NA	NA	NA	NA	NA
Urinary bladder hemorrhage	53	0	—	<0.001^e^	NA	NA	NA	NA	NA	NA	NA	NA
Adhesion	52	2	39.638^f^	<0.001^g^	NA	NA	NA	NA	NA	NA	NA	NA
Musculoskeletal chest pain	52	19	12.139^a^	<0.001^b^	NA	NA	NA	NA	NA	NA	NA	NA
Hemorrhage urinary tract	51	1	41.347^f^	<0.001^g^	NA	NA	NA	NA	NA	NA	NA	NA
Hydronephrosis	50	0	—	<0.001^e^	NA	NA	NA	NA	NA	NA	NA	NA
Abscess	47	36	0.541^a^	0.462^b^	NA	NA	NA	NA	NA	NA	NA	NA
Groin pain	47	27	3.538^a^	0.06^b^	NA	NA	NA	NA	NA	NA	NA	NA
Bladder disorder	45	20	7.216^a^	0.007^b^	NA	NA	NA	NA	NA	NA	NA	NA
Osteopenia	44	29	1.726^a^	0.189^b^	NA	NA	NA	NA	NA	NA	NA	NA
Acne	44	124	47.034^a^	<0.001^b^	9	64	59.492^a^	<0.001^b^	NA	NA	NA	NA
Micturition urgency	41	49	1.791^a^	0.181^b^	NA	NA	NA	NA	NA	NA	NA	NA
Dementia Alzheimer’s type	39	1	30.579^f^	<0.001^g^	106	0	NA	<0.001^e^	NA	NA	NA	NA
Aortic aneurysm	38	0	—	<0.001^e^	NA	NA	NA	NA	NA	NA	NA	NA
Aggression	37	38	0.321^a^	0.571^b^	11	40	26.039^a^	<0.001^b^	NA	NA	NA	NA
Sciatica	36	23	1.678^a^	0.195^b^	NA	NA	NA	NA	NA	NA	NA	NA
Bone lesion	31	2	20.998^f^	<0.001^g^	NA	NA	NA	NA	NA	NA	NA	NA
Pituitary hemorrhage	30	0	—	<0.001^e^	NA	NA	NA	NA	NA	NA	NA	NA
Increased appetite	30	65	16.842^a^	<0.001^b^	8	38	29.359^a^	<0.001^b^	NA	NA	NA	NA
Affect lability	30	79	27.504^a^	<0.001^b^	13	29	11.544^a^	0.001^b^	NA	NA	NA	NA
Cold sweat	29	51	8.579^a^	0.003^b^	NA	NA	NA	NA	NA	NA	NA	NA
Apathy	27	31	0.851^a^	0.356^b^	NA	NA	NA	NA	NA	NA	NA	NA
Proctalgia	26	14	2.468^a^	0.116^b^	NA	NA	NA	NA	NA	NA	NA	NA
Anal incontinence	25	8	7.101^a^	0.008^b^	NA	NA	NA	NA	NA	NA	NA	NA
Exercise tolerance decreased	25	18	0.530^a^	0.467^b^	NA	NA	NA	NA	NA	NA	NA	NA
Abscess sterile	19	41	10.546^a^	0.001^b^	NA	NA	NA	NA	NA	NA	NA	NA
Hair growth abnormal	19	35	6.589^a^	0.010^b^	4	51	53.452^f^	<0.001^g^	NA	NA	NA	NA
Fat tissue increased	12	18	1.910^a^	0.167^b^	NA	NA	NA	NA	NA	NA	NA	NA
Neutropenia	—	—	—	—	NA	NA	NA	NA	64	4	1.227^f^	0.268^g^

AEs listed above had significant signal strengths. NA, Not Applicable (for relevant criterias). N: cases of serious/non-serious adverse events. GnRHa, gonadotrophin-releasing hormone analog.

*p*-value <0.05 was considered statistically significant.

^a^ χ2 statistic of the Pearson chi-squared test.

^b^ Proportions were compared using the Pearson χ2 test.

^c^ Z statistic of the Mann–Whitney U test.

^d^ Mann–Whitney U test.

^e^ Proportions were compared using Fisher’s exact test.

^f^ χ2 statistic of the Yates’s correction for continuity.

^g^ Proportions were compared using Yates’s correction for continuity.

### Sensitivity analyses of GnRHas from FAERS

We performed sensitivity analyses (excluding all concomitant medications) of GnRHas from FAERS in Supplementary Table S3. Out of total 312 PTs, 34 PTs showed the significant differences (*p* < 0.05), such as dysuria, pollakiuria, urinary retention, hematuria, and nocturia. Overall, other results were largely similar and consistent with the analysis that included all concomitant medications based on the aROR for GnRHas.

### Signals of same preferred terms from JADER

**FIGURE 3:**
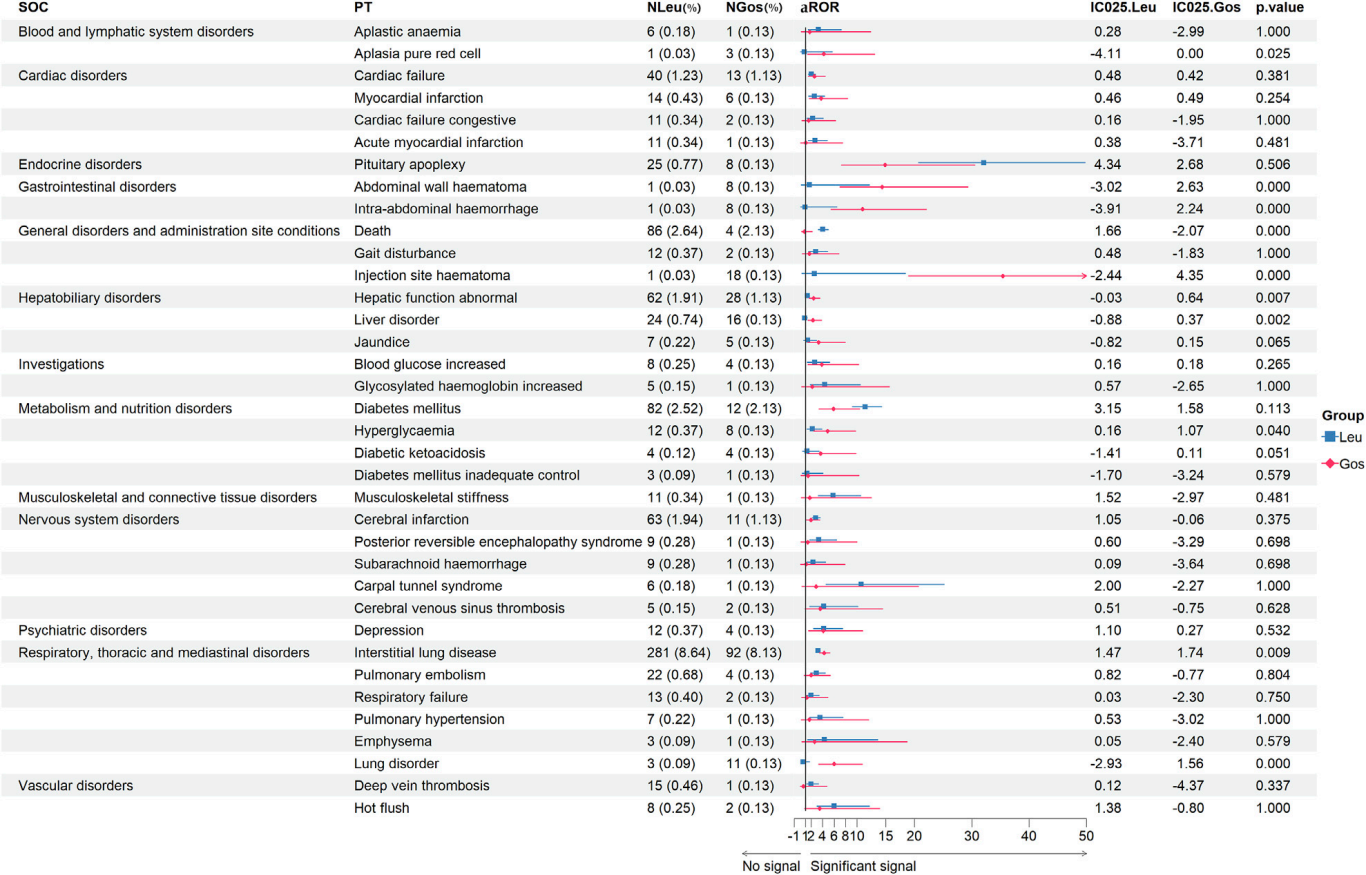
Signal strength of the same adverse events of leuprolide and goserelin at the PT level from JADER databases. -, showed a negative signal (aROR_025_ ≤ 1, and IC_025_ ≤ 0); *p* < 0.05, showed significant difference by Pearson χ^2^ test; IC_025_ > 0 and aROR_025_ > 1, showed a suspicious signal. PT, preferred term; JADER, Japanese Adverse Drug Event Report; SOC, system organ class; JADER, Japanese Adverse Drug Event Report; Leu, leuprolide; Gos, goserelin; N, number of target adverse events; aROR, adjusted reporting odds ratio; IC_025_, adjusted lower limit of 95% confidence interval of the information component of BCPNN; BCPNN, Bayesian confidence propagation neural network.

We collected the same PTs of Leu and Gos from JADER (Figure 3). From the same 36 PTs, we found ten with *p* < 0.05, including death (aROR, 4.03 vs. 0.81; *p* < 0.001), interstitial lung disease (aROR, 3.18 vs. 4.25; *p* = 0.009), injection site hematoma (aROR, 2.53 vs. 35.43; *p* < 0.001), hyperglycemia (aROR, 2.20 vs. 4.86; *p* = 0.040), abdominal wall hematoma (aROR, 1.70 vs. 14.36; *p* < 0.001), abnormal hepatic function (aROR, 1.31 vs. 2.41; *p* = 0.007), intra-abdominal hemorrhage (aROR, 0.91 vs. 10.97; *p* < 0.001), liver disorder (aROR, 0.87 vs. 2.31; *p* = 0.002), pure red cell aplasia (aROR, 0.80 vs. 4.20; *p* = 0.025), and lung disorder (aROR, 0.55 vs. 6.01; *p* < 0.001).

### Clinical prioritization of suspicious PTs

We performed a clinical prioritization of the suspicious PTs (unlisted in instructions) of four GnRHas (Leu, Tri, Gos, and Leu-JADER) (Table 3). Of the 110 unlisted PTs (from Leu, Tri, Gos, and Leu-JADER), 43 with significant signals were categorized as IMEs, with only three representing DMEs: hepatitis fulminant (aROR 4.71, 95% CI 1.76–12.55, from Tri), hepatic necrosis (aROR 3.44, 95% CI 1.29–9.19, from Gos), and aplastic anemia (aROR 3.23, 95% CI 1.45–7.23, from Leu-JADER). Based on the clinical priority score, 41 PTs (Leu), 26 PTs (Tri), 24 PTs (Gos), and 8 PTs (Leu-JADER) were graded as weak. Three PTs (Leu), 2 PTs (Tri), 4 PTs (Gos), and 2 PTs (Leu-JADER) were graded as moderate. Notably, in assessing the relevant evidence, two PTs (loss of libido and urinary tract toxicity caused by Leu), one PT (electrolyte imbalance caused by Tri), and two PTs (anorexia and suicidal ideation caused by Gos) showed a strong level of evidence with “++.”

**TABLE 3:** Signal strength and clinical priority assessing results of suspicious PTs (unlisted in instructions).

SOC	PT	Cases reporting PTs	aROR	IC025	Death (n)	IME/DME	Relevant evidence evaluation	Priority level (score)
Leuprorelin-FAERS								
Cardiac disorders	Heart valve calcification*	4	4.16 (1.55–11.22)	0.29	2	NA	-	Weak (1)
Endocrine disorders	Autoimmune thyroiditis*	19	1.88 (1.20–2.95)	0.14	1	IME	+	Weak (2)
	Hypopituitarism*	13	2.81 (1.63–4.85)	0.55	0	NA	+	Weak (2)
	Hypothalamo-pituitary disorder*	12	3.05 (1.73–5.39)	0.63	0	IME	-	Weak (3)
	Androgen deficiency*	6	4.26 (1.90–9.56)	0.68	0	NA	-	Weak (1)
	Thyroid cyst*	6	2.89 (1.29–6.47)	0.12	0	NA	-	Weak (1)
	Anovulatory cycle*	5	4.91 (2.02–11.94)	0.73	0	NA	-	Weak (1)
	Ectopic ACTH syndrome*	4	6.93 (2.48–19.35)	1.03	2	IME	-	Weak (3)
	Graves disease*	3	4.23 (1.34–13.33)	0.01	0	IME	+	Weak (2)
Eye disorders	Optic disc disorder*	4	3.97 (1.47–10.69)	0.22	0	NA	-	Weak (1)
Gastrointestinal disorders	Anal incontinence*	33	1.54 (1.10–2.17)	0.05	2	NA	-	Weak (1)
	Intestinal polyp*	14	2.74 (1.62–4.63)	0.55	0	NA	-	Weak (2)
	Abdominal fat apron*	8	11.82 (5.65–24.76)	2.35	0	NA	-	Weak (2)
	Pelvic floor dysfunction*	4	6.12 (2.23–16.78)	0.85	0	NA	+	Weak (2)
General disorders and administration site conditions	Gait disturbance*	602	1.12 (1.04–1.22)	0.03	35	NA	+	Weak (2)
Infections and infestations	Bacterial vaginosis*	9	2.22 (1.15–4.29)	0.01	0	NA	-	Weak (1)
	Tracheobronchitis bacterial*	3	6.29 (1.81–21.89)	0.58	0	NA	-	Weak (2)
	Infected lymphocele*	3	4.78 (1.50–15.20)	0.19	0	IME	-	Weak (2)
Investigations	Prostate-specific antigen increased*	1787	45.96 (43.69–48.35)	5.44	178	NA	+	Moderate (4)
	Prostate-specific antigen abnormal*	293	97.58 (84.29–112.97)	6.42	16	NA	+	Moderate (4)
	Luteinizing hormone abnormal*	15	24.63 (12.92–46.95)	3.75	0	NA	-	Weak (2)
	Follicle-stimulating hormone abnormal*	13	19.59 (10.55–36.37)	3.35	0	NA	-	Weak (3)
Metabolism and nutrition disorders	Cachexia*	27	1.82 (1.25–2.66)	0.22	15	IME	-	Weak (2)
Musculoskeletal and connective tissue disorders	Mobility decreased*	372	1.96 (1.77–2.17)	0.80	43	NA	-	Weak (2)
	Dupuytren’s contracture*	7	2.62 (1.24–5.51)	0.09	0	IME	-	Weak (2)
	Soft tissue necrosis*	6	2.75 (1.23–6.16)	0.05	0	IME	-	Weak (2)
Nervous system disorders	Sciatica*	59	1.52 (1.18–1.96)	0.17	1	NA	+	Weak (2)
	Monoplegia*	21	1.67 (1.09–2.57)	0.01	1	NA	-	Weak (1)
	Cerebrovascular spasm*	4	4.75 (1.76–12.84)	0.48	0	NA	+	Weak (1)
Psychiatric disorders	Loss of libido*	154	6.38 (5.44–7.48)	2.41	2	NA	++	Moderate (4)
	Vaginismus*	7	10.58 (4.82–23.21)	2.10	0	NA	-	Weak (2)
	Gender dysphoria*	7	10.53 (4.80–23.08)	2.09	0	NA	-	Weak (2)
Renal and urinary disorders	Urinary tract toxicity*	7	12.87 (5.37–30.80)	2.38	0	NA	++	Weak (2)
	Bladder perforation*	5	3.37 (1.39–8.14)	0.19	1	IME	-	Weak (2)
	Follicular cystitis*	3	6.56 (1.70–25.39)	0.65	0	IME	-	Weak (3)
Reproductive system and breast disorders	Uterine spasm*	39	6.67 (4.86–9.17)	2.21	0	NA	-	Weak (3)
	Prostatomegaly*	39	3.47 (2.53–4.76)	1.26	5	NA	-	Weak (2)
	Pelvic fluid collection*	6	2.75 (1.23–6.15)	0.04	0	NA	-	Weak (1)
	Reproductive toxicity*	5	9.07 (3.49–23.56)	1.62	0	IME	-	Weak (3)
Respiratory, thoracic and mediastinal disorders Vascular disorders	Chronic respiratory disease*	5	4.06 (1.67–9.83)	0.46	1	NA	-	Weak (1)
	Hemorrhage*	424	1.52 (1.38–1.67)	0.44	14	NA	+	Weak (2)
	Aortic aneurysm*	38	1.69 (1.23–2.33)	0.22	6	NA	+	Weak (1)
	Aneurysm*	26	1.84 (1.25–2.71)	0.23	7	NA	+	Weak (1)
	Aortic dilatation*	8	2.49 (1.24–4.99)	0.10	0	NA	-	Weak (1)
Triptorelin-FAERS								
Cardiac disorders	Acute coronary syndrome*	8	4.67 (2.34–9.35)	1.01	0	IME	+	Weak (2)
Eye disorders	Retinal vein thrombosis*	3	5.7 (1.83–17.72)	0.44	0	NA	-	Weak (2)
	Choroidal neovascularisation*	4	7.3 (2.73–19.51)	1.10	0	IME	-	Weak (3)
Gastrointestinal disorders	Ascites*	19	4.01 (2.55–6.29)	1.23	0	IME	+	Weak (3)
	Peritoneal hemorrhage*	3	4.67 (1.50–14.50)	0.15	0	NA	-	Weak (1)
Hepatobiliary disorders	Hepatitis fulminant*	4	4.71 (1.76–12.55)	0.47	4	DME	-	Weak (3)
Immune system disorders	Overlap syndrome*	3	6.21 (1.99–19.36)	0.57	0	NA	-	Weak (2)
Infections and infestations	Impetigo*	3	4.92 (1.58–15.27)	0.23	0	NA	-	Weak (1)
	Appendicitis*	5	3.07 (1.28–7.39)	0.06	0	IME	-	Weak (2)
	Erysipelas*	5	4.41 (1.84–10.61)	0.58	0	IME	-	Weak (2)
Injury, poisoning and procedural complications	Fracture*	9	2.87 (1.49–5.52)	0.38	0	NA	-	Weak (1)
	Femoral neck fracture*	13	10.75 (6.23–18.54)	2.49	0	IME	-	Moderate (4)
Investigations	Luteinizing hormone increased*	5	10.21 (4.21–24.80)	1.79	0	NA	+	Weak (2)
	Luteinizing hormone decreased*	6	12.32 (5.45–27.86)	2.21	0	NA	+	Weak (2)
	Prostate-specific antigen increased*	37	14.67 (10.62–20.27)	3.33	4	NA	+	Weak (3)
	Thyroid hormones increased*	3	5.48 (1.76–17.04)	0.39	0	NA	-	Weak (2)
Metabolism and nutrition disorders	Electrolyte imbalance*	6	3.06 (1.38–6.82)	0.20	5	NA	++	Weak (1)
Musculoskeletal and connective tissue disorders	Muscle atrophy*	7	3.39 (1.61–7.11)	0.46	0	NA	-	Weak (1)
	Osteoporosis*	22	3.39 (2.23–5.16)	1.05	0	NA	+	Weak (2)
	Polymyositis*	4	5.87 (2.20–15.67)	0.79	0	IME	-	Weak (3)
	Polyneuropathy*	6	3.04 (1.36–6.76)	0.19	1	IME	-	Weak (2)
Nervous system disorders	Dementia Alzheimer’s type*	106	56.47 (46.56–68.47)	5.50	0	IME	-	Moderate (5)
	Benign intracranial hypertension*	4	6.22 (2.33–16.59)	0.87	0	NA	-	Weak (2)
Psychiatric disorders	Depression suicidal*	4	4.41 (1.65–11.75)	0.37	0	IME	-	Weak (2)
	Intentional self-injury*	8	2.41 (1.21–4.83)	0.06	0	IME	-	Weak (2)
Renal and urinary disorders	Hydronephrosis*	5	3.42 (1.42–8.23)	0.21	2	IME	-	Weak (2)
	Hypertonic bladder*	4	4.98 (1.87–13.29)	0.55	0	NA	-	Weak (1)
Vascular disorders	Hypertensive crisis*	7	3.43 (1.64–7.20)	0.48	0	IME	-	Weak (2)
Goserelin-FAERS								
Blood and lymphatic system disorders	Lymphadenopathy*	18	2.47 (1.55–3.92)	0.51	3	NA	-	Weak (2)
	Abdominal lymphadenopathy*	5	8.42 (3.49–20.31)	1.51	1	NA	-	Weak (2)
Cardiac disorders	Coronary artery dissection*	3	5.28 (1.70–16.43)	0.33	1	IME	-	Weak (3)
Ear and labyrinth disorders	Hyperacusis*	5	4.42 (1.84–10.62)	0.58	0	NA	-	Weak (1)
Endocrine disorders	Acute thyroiditis*	3	6.62 (2.10–20.84)	0.66	0	NA	-	Weak (2)
Gastrointestinal disorders	Ascites*	24	3.84 (2.57–5.74)	1.26	1	IME	+	Weak (3)
	Abdominal wall hematoma*	12	13.81 (7.83–24.38)	2.81	0	IME	-	Moderate (4)
	Intra-abdominal hematoma*	11	15.28 (8.43–27.68)	2.91	0	IME	-	Moderate (4)
	Intra-abdominal hemorrhage*	10	10.53 (5.66–19.61)	2.32	0	IME	-	Moderate (4)
Hepatobiliary disorders	Jaundice*	15	2.52 (1.52–4.18)	0.46	1	NA	+	Weak (2)
	Hepatotoxicity*	12	2.67 (1.52–4.71)	0.44	0	IME	-	Weak (3)
	Hepatic necrosis*	4	3.44 (1.29–9.19)	0.02	1	DME	-	Weak (3)
Investigations	Prostate-specific antigen increased*	51	15.76 (11.96–20.76)	3.51	2	NA	+	Moderate (4)
	Alkaline phosphatase increased*	17	3.03 (1.88–4.87)	0.78	1	NA	+	Weak (2)
Metabolism and nutrition disorders	Anorexia*	8	2.33 (1.17–4.66)	0.01	0	NA	++	Weak (1)
Musculoskeletal and connective tissue disorders	Muscle atrophy*	12	4.45 (2.53–7.85)	1.18	2	NA	-	Weak (2)
	Trigger finger*	6	5.12 (2.30–11.41)	0.94	0	NA	-	Weak (2)
Nervous system disorders	Cognitive disorder*	23	2.46 (1.63–3.70)	0.60	0	NA	-	Weak (2)
	Dementia Alzheimer’s type*	6	2.75 (1.23–6.12)	0.04	1	IME	-	Weak (2)
	Paraplegia*	4	4.14 (1.55–11.04)	0.28	0	IME	-	Weak (2)
	Cerebral venous sinus thrombosis*	3	5.71 (1.83–17.77)	0.44	0	IME	-	Weak (3)
	Suicidal ideation*	30	1.61 (1.13–2.31)	0.08	0	IME	++	Weak (2)
	Urinary retention*	15	2.2 (1.33–3.66)	0.27	2	IME	+	Weak (3)
	Hematuria*	15	2.03 (1.23–3.38)	0.15	2	NA	+	Weak (2)
	Kidney enlargement*	3	4.83 (1.56–15.02)	0.20	0	NA	-	Weak (1)
Reproductive system and breast disorders	Amenorrhoea*	21	5.48 (3.57–8.42)	1.72	0	NA	+	Weak (3)
	Pleural effusion*	32	2.54 (1.79–3.59)	0.75	4	NA	+	Weak (2)
	Pneumonitis*	11	2.13 (1.18–3.84)	0.06	0	IME	-	Weak (3)
Leuprorelin-JADER								
Blood and lymphatic system disorders	Aplastic anemia*	6	3.23 (1.45–7.23)	0.28	0	DME	-	Weak (3)
Eye disorders	Retinal vein occlusion*	10	7.61 (4.06–14.28)	1.85	0	IME	+	Moderate (4)
Gastrointestinal disorders	Mesenteric artery thrombosis*	3	5.3 (1.67–16.85)	0.34	1	IME	-	Weak (3)
Investigations	Prostate-specific antigen increased*	4	7.01 (2.54–19.31)	1.04	0	NA	+	Weak (2)
Musculoskeletal disorders	Trigger finger*	4	8.35 (2.72–25.62)	1.30	0	NA	-	Weak (2)
Nervous system disorders	Cerebral vasoconstriction syndrome*	10	12.82 (6.73–24.41)	2.60	0	IME	+	Moderate (4)
	Posterior encephalopathy syndrome*	9	3.33 (1.73–6.42)	0.60	0	IME	+	Weak (2)
	Multiple sclerosis*	4	4.81 (1.78–12.95)	0.50	0	IME	-	Weak (2)
Respiratory, thoracic, and mediastinal disorders	Respiratory failure*	13	1.96 (1.13–3.38)	0.03	12	IME	+	Weak (2)
	Acute respiratory failure*	6	3.05 (1.37–6.82)	0.19	5	IME	-	Weak (2)

PTs, preferred terms; aROR, adjusted reporting odds ratio; CI, confidence interval; IME, important medical event; DME, designated medical event; NA, not applicable (for relevant criteria); n, number of cases. *, showed the unlisted AEs (adverse events) in the dispensatories of GnRHas (Gonadotrophin-releasing hormone analogs).

++: AEs are mainly from the FDA prescribing information, the Summary of Product Characteristics of GnRHas posted by the MHRA, Phase 2/3 RCTs, or systematic reviews, with biological plausibility. +: AEs are mainly from other clinical trials, observational studies, or case reports/series with potential biological plausibility. -, AEs only emerging from disproportionality analyses.

### Signal profiles of different GnRHas (top 30 of IC_025_)

**FIGURE 4:**
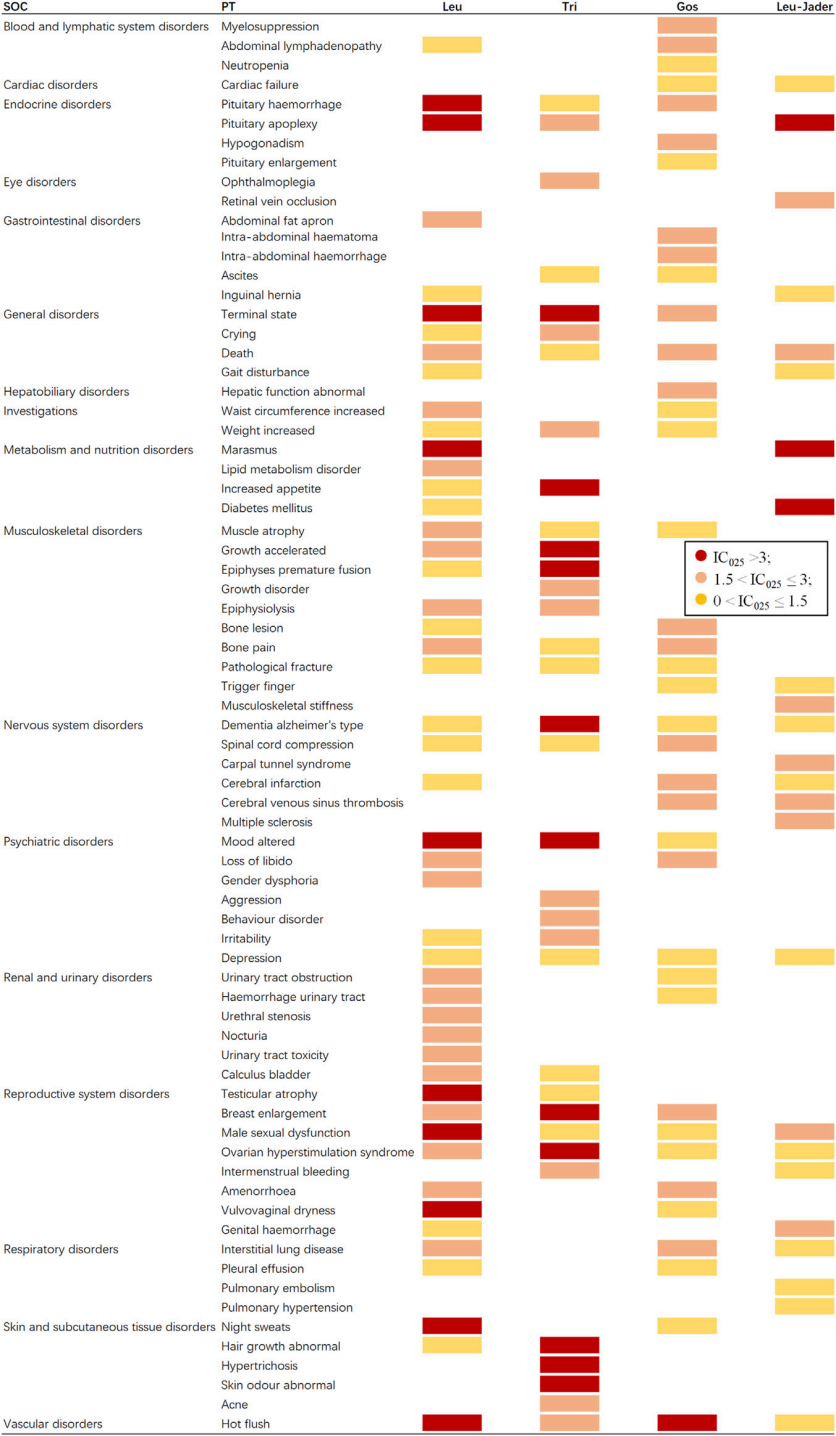
Signal profiles of different GnRHas from FAERS and JADER databases (top 30 based on IC_025_ of each GnRHas). GnRHas, gonadotropin-releasing hormone analogs; FAERS, FDA Adverse Event Reporting System; JADER, Japanese Adverse Drug Event Report; SOC, system organ class; PT, preferred term; Leu, leuprolide; Tir, triptorelin; Gos, goserelin; IC_025_, adjusted the lower limit of 95% confidence interval of the information component of BCPNN; BCPNN, Bayesian confidence propagation neural network. IC_025_ > 3.0 indicates a strong signal; 1.5 < IC_025_ ≤ 3.0 indicates a medium-intensity signal; 0 < IC_025_ ≤ 1.5 indicates a weak-intensity signal.

If data mining was conducted only for the same PTs, some important signals might be ignored. Therefore, the signal spectrum of each GnRHa is shown in Figure 4, where the top 30 of the 95% confidence intervals (95%CI) of IC (IC_025_) is regarded as an indicator; whole signals of different GnRHas are displayed in Supplementary Table S2. If IC_025_ > 3.0, there is a strong signal; 1.5 < IC_025_ ≤ 3.0, indicates a medium intensity signal; 0 < IC_025_ ≤ 1.5, indicates a weak intensity signal (Li Sun et al., 2019; Zou et al., 2023b).

As shown in Figure 4 (excluding PTs caused by indications, operations, and unqualified products), Tri and Leu (FAERS) presented the greatest spectrum (IC_025_ > 3.0), with a total of 11 and 10 strong GnRHas-induced signals (12 and 20 medium signals) detected, ranging from endocrine disorders to vascular disorders, following by Gos (one strong and 18 medium signals) and Leu-JADER (three strong and nine medium signals). In Figure 4, the suspicious PTs of Leu were obviously concentrated in endocrine disorders (pituitary hemorrhage [IC_025_ 3.74] and pituitary apoplexy [IC_025_ 3.14]), metabolism and nutrition disorders (marasmus [IC_025_ 3.14], lipid metabolism disorder [IC_025_ 2.63], increased appetite [IC_025_ 2.53], and diabetes mellitus [IC_025_ 1.42]), renal and urinary disorders (urinary tract obstruction [IC_025_ 2.91], urinary tract hemorrhage [IC_025_ 2.32], urethral stenosis [IC_025_ 2.51], nocturia [IC_025_ 2.42], urinary tract toxicity [IC_025_ 2.38], and calculus bladder [IC_025_ 1.97]). Interestingly, from FAERS, some suspicious PTs of Tri differed from Leu (FAERS) in nervous system disorders (Alzheimer’s type dementia [IC_025_ 5.50 vs. 2.09]), musculoskeletal disorders (epiphyses premature fusion [IC_025_ 4.89 vs. 0.93]), and skin disorders (abnormal hair growth [IC_025_ 4.73 vs. 1.01], hypertrichosis [IC_025_ 3.63], abnormal skin odor [IC_025_ 3.49], and acne [IC_025_ 2.19]). Notably, hot flush was the only PT significantly related to all GnRHas from FAERS with markedly strong intensity.

### Onset time of GnRHas-related AEs

Because the effective onset-time of Leu-JADER was too short to calculate, we only detected the GnRHas (Leu, Tri, and Gos) from the FAERS database. We collected the onset times of GnRHas-associated AEs from FAERS (Supplementary Figure S2). The onset times of adverse events of Leu and Tri were highly similar. The median onset time of GnRHas (Leu, Tri, and Gos) was 23 days (0, 298), 22 days (0, 181), and 217 days (29, 706), as median (Q1, Q3).

## Discussion

To the best of our knowledge, we were the first to mine GnRHas data from the FAERS and JADER databases. Juan Tamargo et al. achieved greater understanding of the differences in the genetic variants of drug-metabolizing enzymes (DMEs) and transporters that determine the differences in the exposure, efficacy, and safety of cardiovascular drugs between races/ethnicities(Tamargo et al., 2022). Our study showed that the most commonly reported and newest signals of GnRHas were found at the SOC and PT levels in two databases.

In our disproportionality analysis, many organs or tissues could have been under the influence of GnRHas at SOC levels. Tri (FAERS) seemed to have the broadest and strongest signals of SOC, such as for reproductive system disorders (ROR 8.44, IC_025_ 2.92), product issues (aROR 6.97, IC_025_ 2.67), endocrine disorders (aROR 4.46, IC_025_ 1.67), psychiatric disorders (aROR 1.99, IC_025_ 0.88), social circumstances (aROR 3.72, IC_025_ 1.39), and skin disorders (aROR 1.17, IC_025_ 0.07). Unlike JAERS, Leu from JADER had also the broadest aROR of SOC, although many of the values are not the largest and had the characteristic SOCs, such as cardiac disorders (aROR 1.48, IC_025_ 0.29) and eye disorders (aROR 2.53, IC_025_ 0.63). The reason for this might be that most reports were by medical personnel in Japan and most by public in the United States (Table 1). For example, for hot flush in Figure 4, three GnRHas (Leu, Tri, and Gos from FAERS) showed strong signals (IC_025_, 5.34, 2.98, and 3.81) but Leu (JADER) showed a weak signal (IC_025_, 1.38).

Furthermore, we found that some PTs might result in serious outcomes, with statistically significant differences observed between severe and non-severe cases (*p* < 0.001) in different GnRHas, such as hot flush, osteoporosis, and dementia. A prospective, single-center, single-arm, open label study of the long-term use of Tri showed an increased risk of developing osteopenia after 12 months of treatment (Alshehre et al., 2020). A 4-year interventional case–control study reported a case of metatarsal fracture (Dotremont et al., 2023). Similarly, we also found the clinical priority for femoral neck fracture caused by Tri (aROR 10.75, 95% CI 6.23–18.54) was moderate.

It is noteworthy that the aROR of dementia (Alzheimer’s type, *n* = 106) was 56.47 (95%, 46.56–68.47) with IC_025_ (5.50, a strong signal) caused by Tri. The clinical priority of the PT showed a moderate (Zondervan et al., 2020) signal. A cohort study of 23,651 patients with newly diagnosed prostate cancer showed the use of antiandrogen monotherapy was associated with an increased risk of dementia or Alzheimer’s disease (AD) (Huang et al., 2020).

Interestingly, even for the same PTs, the aROR values of different GnRHas were quite different. In Figures 2 and 3, only positive signals showed in the forest plot. A meta-analysis of the use of GnRHas for the treatment of endometriosis revealed that the most frequently reported adverse events included vaginal dryness, hot flushes, headaches, muscle cramps (myalgia), sleep disturbance (insomnia), altered libido, weight gain, bone loss, and acne (Veth et al., 2023; Zheng et al., 2023).

A clinical study of leuprolide acetate 6-month depot showed that eight subjects in the study encountered AEs that were associated with diabetes mellitus (*n* = 2), elevations in serum glucose (*n* = 5), or hypoglycemia (*n* = 1) (Spitz et al., 2012). Another study involving girls with idiopathic CPP treated with GnRHas also demonstrated a deterioration in glucose metabolism during GnRHas treatment, with complete restoration observed afterward; this effect was independent of pre-treatment body mass index (BMI) (Bruzzi et al., 2022). Coincidentally, we found that only leuprolide had a positive signal for diabetes mellitus (aROR 1.26, 95% CI 1.12–1.42, IC_025_ 0.14) in FAERS. Interestingly, in Supplementary Table S2, compared with FAERS, leuprolide (JADER) had stronger signals in metabolism disorders such as increased blood glucose (aROR 2.59 vs. no-significant), increased blood triglycerides (aROR 3.48 vs. no-significant), diabetes mellitus (aROR 11.43 vs. 1.26), and marasmus (aROR 45.03 vs. 14.58). An observational study of 37,443 population-based men showed that androgen deprivation therapy with GnRHa was associated with an increased risk of diabetes (Keating et al., 2010). In a mechanistic study of fat accumulation, Dr Hefeng Huang found that FSH downregulated aquaporin 7 (AQP7) expression and glycerol efflux function in mature adipocytes of post-menopausal women and ovariectomized (OVX) mice (Chen et al., 2020).

However, although Leu had the broadest positive signals in same PTs (Figure 4), Tri had the greatest number of strong signals (aROR ≥ 10) for the following PTs: Alzheimer’s type dementia (aROR 56.47, 95% CI 46.56–68.47), abnormal hair growth (aROR 36.21, 95% CI 27.75–47.27), terminal state (aROR 24.37, 95% CI 17.55–33.84), altered mood (aROR 23.41, 95% CI 19.31–28.39), abnormal skin odor (aROR 18.86, 95% CI 12.14–29.28), increased appetite (aROR 15.77, 95% CI 11.8–21.08), mood swings (ROR 15.31, 95% CI 12.33–19.01), pituitary apoplexy (aROR 14.54, 95% CI 6.72–31.45), and personality change (aROR 10.36, 95% CI 6.68–16.08).

Hormone therapy was not perfect: Wang et al. (2023) discovered that androgen deprivation therapy (ADT), including GnRHas, induced SPP1+ myofibroblastic cancer-associated fibroblasts (myCAFs), which proved to be crucial stromal constituents that drove the development of castration-resistant prostate cancer (CRPC). In ovarian hyperstimulation syndrome (OHSS), a rare but potentially life-threatening condition (for every 55 patients with a long GnRHa protocol, one would be hospitalized because of OHSS), some evidences indicates that the routine use of GnRH-ant instead of GnRHa during ovarian stimulation drastically reduces the relative risk of OHSS (Kolibianakis et al., 2006; Al-Inany et al., 2007; Griesinger, 2010).

Extended periods of high hormone concentration might potentially lead to hormone-related adverse events (AEs). The reason for the difference might be the different signaling pathways, structure, and dosage form of GnRHas. Robert P Millar et al. (2008) found that the effects of various GnRHas were notably distinct in relation to their impact on the pituitary gonadotrope, as they engage different signaling pathways (Millar et al., 2008). The GnRH receptor can adopt different conformations, each exhibiting varying selectivity for GnRHas and intracellular signaling protein complexes (Millar et al., 2008).

Even if both GnRHas acted on the reproductive system (Supplementary Table S2), their effects were different. In the reproductive system, prostatic GnRH- receptors appeared to be different from the pituitary, endometrium, and ovary, which showed a lower binding affinity for GnRH and its analogs (Limonta et al., 1992; Dondi et al., 1994; Casati et al., 2023). Thus, the adverse effects of GnRHa on women and men might be different. However, it is worth noting that when concomitant medications were removed in a sensitivity analysis, the aROR of GnRHa from FAERS were largely in keeping with the results from the primary analysis (Supplementary Table S3).

Finally, we collected the onset time of GnRHas in Supplementary Figure S2. The onset-time curves of adverse events caused by Leu and Tri were highly similar, as median (Q1, Q3) (23 days [0, 298] and 22 days [0, 181]). The results might be caused by the similar protein structure of Leu and Tri, which were derived from the native GnRH by substitution of a L-glycine in position 6 (Warner et al., 1983; Lahlou, 2005).

Our study also had certain limitations. First, because of the concomitant medications, missing, incomplete and repetitive reports, disproportionality analysis alone cannot prove causation or measure incidence, and specific limitations, including confounding, reporting bias and efforts to mitigate them. Second, the number of total cases had large differences between the two databases. JADER is a database limited to individual case reports, which reports serious cases individually, while FAERS also includes periodic reports that collectively report non-serious cases collected over a period of time (Noguchi et al., 2021). However, we applied the statistical shrinkage transformation for the sake of robustness to minimize this bias as much as possible. Third, in this study, we did not conduct sensitivity analyses on the influence of the indications of GnRHas. Notably, even if the number of FAERS reports is limited to data from Japan and the United States, the available Japanese data account for less than 5% of the total United States data (Nawa et al., 2021).

Due to the distinct signaling pathways, further investigation into GnRH analogs (GnRHas) held significant value for the fields of pharmacovigilance and pharmacodynamics, especially diverse racial populations. We anticipated that our analysis, utilizing spontaneous adverse drug event report databases, will serve as a valuable contribution to future studies in this domain.

## Conclusion

This is the first study to analyze GnRHas from the FAERS and JADER databases comprehensively and systematically. The continuous monitoring of drug safety profiles in real-world scenarios is imperative. Moreover, our study provided the differences of GnRHas from FAERS and JADER.

## Data Availability

Publicly available datasets were analyzed in this study. These data can be found at: https://fis.fda.gov/extensions/FPD-QDE-FAERS/FPD-QDE-FAERS.html and https://www.info.pmda.go.jp/fukusayoudb/CsvDownload.

## Supplementary material

SUPPLEMENTARY FIGURE 1 Process of searching GnRHas-associated adverse events from the FAERS and JADER databases. GnRHas, gonadotropin-releasing hormone analogs; FAERS, FDA Adverse Event Reporting System; JADER, Japanese Adverse Drug Event Report.

SUPPLEMENTARY FIGURE 2 Time to onset of different GnRHas-related AEs from the FAERS databases (Unit: days). GnRHas, gonadotropin-releasing hormone analogs; AEs, adverse events; FAERS, FDA Adverse Event Reporting System; Leu, leuprolide; Tir, triptorelin; Gos, goserelin. The median onset time of GnRHas (Leu, Tri, and Gos) was 23 days (0, 298), 22 days (0, 181), and 217 days (29, 706), as median (Q1, Q3).
