# Supplementary material for: A disproportionality analysis of adverse events caused by GnRHas from the FAERS and JADER databases
Source: Front Pharmacol. 2024 Jul 4;15:1392914. doi: 10.3389/fphar.2024.1392914 (PMC11254796; doi:10.3389/fphar.2024.1392914)
Supplement: Supplementary file 3 [file Table1.DOCX]

**Supplementary Table 1** Summary of major algorithms, MeSH of GnRHas used for signal detection, and a rating scale assessing clinical priority.

|  | Reports with the suspected adverse effect | Reports without the suspected adverse effect |
| --- | --- | --- |
| Reports with the suspected drug | a | b |
| Reports without the suspected drug | c | d |

| Algorithms | Equation | Criteria |
| --- | --- | --- |
| Adjusted ROR | N_expected_= N_drug_ × N_event_/N_total_ | lower limit of 95% CI>1, N_observed_≥3 |
|  | ROR= (N_observed_ + 0.5)/ (N_expected_ + 0.5) |  |
|  | 95% CI=e^ln (ROR) ± 1.96(1/a+1/b+1/c+1/d) ^0.5^ |  |
| Adjusted BCPNN | IC= log_2_ ((N_observed_ + 0.5)/ (N_expected_ + 0.5)) | IC_025_>0 |
|  | IC_025_= IC − 3.3 × (N_observed_ + 0.5) ^−0.5^ − 2 × (N_observed_ + 0.5) ^−1.5^ |  |
| ROR | ROR=ad/bc | lower limit of 95% CI>1, a≥3 |

| **GnRHas** | **MeSH (generic names and trade names)** |
| --- | --- |
| Leuprorelin | Leuprorelin, Enantone, Leuprolide, Lupron, A43818, TAP144 |
| Triptorelin | Triptorelin, CL118532, Trelstar, Decapeptyl, AY25650, Decapeptyl Depot |
| Goserelin | ICI 118630, ICI118630, Zoladex, Goserelin |

A rating scale assessing clinical priority of disproportionality signals.

| **Assessment items** | **2 points** | **1 point** | **0 point** |
| --- | --- | --- | --- |
| Number of target events | >50 | 10-50 | <10 |
| ROR | >5 | 2-5 | 1-2 |
| Mortality proportion | >50% | 25-50% | <25% |
| IMEs or DMEs | DME | IME | None |
| Relevant evidence evaluation | ++ | + | **-** |

MeSH: medical subject headings; GnRHas: Gonadotrophin releasing hormone analogues; adjusted ROR: adjusted reporting odds ratio; N_expected_: the expected number of records of target drug adverse events (AEs); N_drug_: the total number of records of the target drug; N_event_: the total number of records of target AEs, and N_total_: the total number of records in the whole database; N_observed_: the observed number of records of target drug AEs; 95% CI: the 95% two-sided of confidence interval; Adjusted BCPNN: Adjusted Bayesian confidence propagation neural network; IC: information component of BCPNN; IC_025_: the lower limit of the 95% two-sided CI of the IC; a: the number of reports with suspect adverse events (AEs) of the suspect drug; b: the number of reports with all other ADEs of the suspect drug; c: the number of reports with the suspect AEs of all other drugs; d: the number of reports with all other AEs of all other drugs; ROR: reporting odds ratio; CI: confidence interval.

Mortality proportion: percentage of cases in which death was reported as an outcome in the overall cases report for a particular adverse event. IMEs and DMEs are developed and updated by EMA (European Medicines Agency, 2022). ++: AEs are mainly from the FDA Prescribing Information, the Summary of Product Characteristics of GnRHas posted by the MHRA, Phase 2/3 RCTs, or systematic reviews, with biological plausibility. +: AEs are mainly from other clinical trials, observational studies, or case reports/series with potential biological plausibility. -: AEs only emerging from disproportionality analyses.

AEs, Adverse Events; DMEs, Designated Medical Events; IMEs, Important Medical Events; MHRA, Medicine and Healthcare Products Regulatory Agency; RCTs, Randomized Controlled Trials; ROR, Reporting odds ratio.
