# Supplementary figures and images for: A disproportionality analysis of adverse events caused by GnRHas from the FAERS and JADER databases
Source: Front Pharmacol. 2024 Jul 4;15:1392914. doi: 10.3389/fphar.2024.1392914 (PMC11254796; doi:10.3389/fphar.2024.1392914)

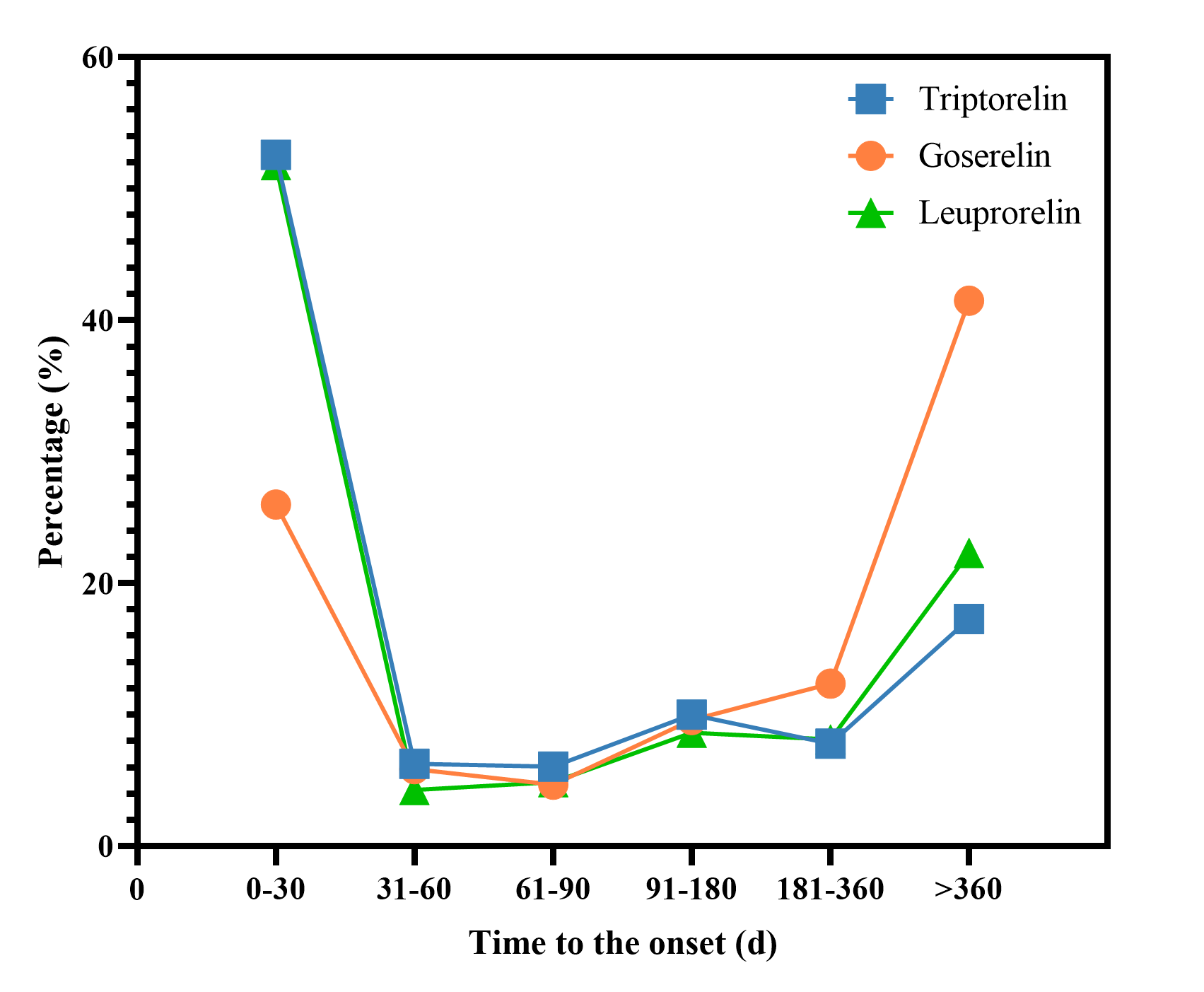

Supplement: Supplementary file 4 [file Image2.TIF]

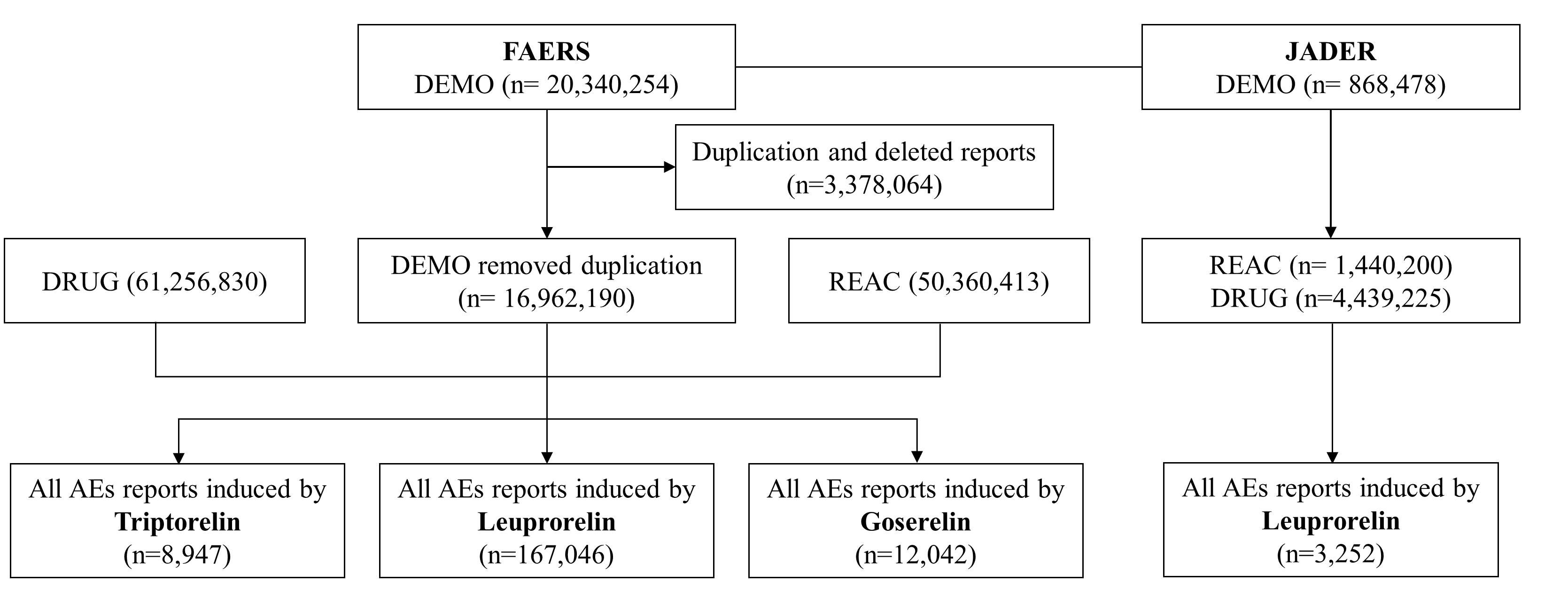

Supplement: Supplementary file 5 [file Image1.TIF]
