# Supplementary material for: A disproportionality analysis of adverse events caused by GnRHas from the FAERS and JADER databases
Source: Front Pharmacol. 2024 Jul 4;15:1392914. doi: 10.3389/fphar.2024.1392914 (PMC11254796; doi:10.3389/fphar.2024.1392914)
Supplement: Supplementary file 6 [file Table4.DOCX]

**Supplement Table 4** the significant PT of leuprorelin and goserelin from FAERS and JADER databases (p<0.05).

| **SOC** | **PT** | **Database** | **aROR Leuprorelin** | **aROR**  **Goserelin** | **IC025-Leu** | **IC025-Gos** | **p** |
| --- | --- | --- | --- | --- | --- | --- | --- |
| Blood and lymphatic system disorders | Aplasia pure red cell | JADER | 0.80 (0.11-5.66) | 4.20 (1.35-13.08) | -4.11 | 0.00 | 0.025 |
| Cardiac disorders | Acute myocardial infarction | JADER | 2.65 (1.46-4.80) | 1.05 (0.15-7.48) | 0.38 | -3.71 | 0.481 |
|  | Myocardial infarction | JADER | 2.57 (1.52-4.36) | 3.74 (1.67-8.36) | 0.46 | 0.49 | 0.254 |
|  | Cardiac failure | JADER | 2.01 (1.47-2.74) | 2.57 (1.49-4.45) | 0.48 | 0.42 | 0.381 |
| Endocrine disorders | Pituitary Haemorrhage | FAERS | 20.40 (13.99-29.75) | 14.87 (7.36-30.02) | 3.74 | 2.68 | <0.01 |
| Gastrointestinal disorders | Abdominal wall haematoma | JADER | 1.70 (0.24-12.16) | 14.36 (7.03-29.32) | -3.02 | 2.63 | 0.000 |
|  | Abdominal Distension | FAERS | 1.51 (1.37-1.66) | 1.72 (1.24-2.40) | 0.43 | 0.22 | 0.47 |
|  | Intra-abdominal haemorrhage | JADER | 0.91 (0.13-6.50) | 10.97 (5.43-22.13) | -3.91 | 2.24 | 0.000 |
| General disorders and administration site conditions | Terminal State | FAERS | 29.51 (27.04-32.19) | 9.49 (5.90-15.29) | 4.74 | 2.43 | <0.01 |
|  | **Death** | **JADER** | **4.03 (3.25-5.00)** | **0.81 (0.30-2.17)** | **1.66** | **-2.07** | **0.000** |
|  | **Death** | **FAERS** | **2.98 (2.91-3.06)** | **7.90 (7.46-8.37)** | **1.54** | **2.89** | **<0.01** |
|  | Injection site haematoma | JADER | 2.53 (0.35-18.45) | 35.43 (18.93-66.32) | -2.44 | 4.35 | 0.000 |
| Hepatobiliary disorders | Jaundice | JADER | 1.40 (0.66-2.94) | 3.29 (1.36-7.92) | -0.82 | 0.15 | 0.065 |
|  | Hepatic function abnormal | JADER | 1.31 (1.02-1.69) | 2.41 (1.65-3.51) | -0.03 | 0.64 | 0.007 |
|  | Liver disorder | JADER | 0.87 (0.58-1.31) | 2.31 (1.41-3.79) | -0.88 | 0.37 | 0.002 |
| Investigations | Blood glucose increased | JADER | 2.59 (1.29-5.20) | 3.85 (1.44-10.30) | 0.16 | 0.18 | 0.265 |
| Metabolism and nutrition disorders | Diabetes mellitus | JADER | 11.43 (9.15-14.27) | 5.90 (3.33-10.44) | 3.15 | 1.58 | 0.113 |
|  | Hyperglycaemia | JADER | 2.20 (1.25-3.89) | 4.86 (2.42-9.77) | 0.16 | 1.07 | 0.040 |
|  | Diabetic ketoacidosis | JADER | 1.28 (0.48-3.41) | 3.66 (1.37-9.80) | -1.41 | 0.11 | 0.051 |
| Musculoskeletal and connective tissue disorders | Musculoskeletal stiffness | JADER | 5.83 (3.21-10.60) | 1.75 (0.25-12.49) | 1.52 | -2.97 | 0.481 |
|  | Bone Pain | FAERS | 3.64 (3.36-3.95) | 4.04 (3.05-5.35) | 1.73 | 1.54 | 0.41 |
|  | Pathological Fracture | FAERS | 2.02 (1.40-2.91) | 5.61 (2.80-11.23) | 0.4 | 1.28 | <0.01 |
|  | Osteoporosis | FAERS | 1.68 (1.46-1.94) | 2.34 (1.51-3.63) | 0.51 | 0.48 | 0.16 |
|  | Muscular Weakness | FAERS | 1.64 (1.50-1.79) | 2.07 (1.55-2.75) | 0.57 | 0.56 | 0.13 |
|  | Bone Disorder | FAERS | 1.62 (1.32-2.00) | 2.37 (1.27-4.40) | 0.35 | 0.17 | 0.25 |
|  | Arthralgia | FAERS | 1.60 (1.52-1.68) | 1.32 (1.09-1.60) | 0.6 | 0.08 | 0.07 |
| Nervous system disorders | Cerebral infarction | JADER | 2.78 (2.16-3.56) | 1.95 (1.08-3.54) | 1.05 | -0.06 | 0.375 |
|  | Spinal Cord Compression | FAERS | 2.46 (1.70-3.57) | 6.54 (3.27-13.10) | 0.67 | 1.5 | <0.01 |
|  | Cerebral Infarction | FAERS | 1.66 (1.39-2.00) | 2.99 (1.83-4.89) | 0.43 | 0.74 | <0.01 |
|  | Dementia Alzheimer's Type | FAERS | 1.54 (1.13-2.09) | 2.75 (1.23-6.12) | 0.09 | 0.04 | 0.16 |
| Psychiatric disorders | Mood Swings | FAERS | 9.61 (8.99-10.27) | 2.02 (1.20-3.42) | 3.15 | 0.11 | <0.01 |
|  | Libido Decreased | FAERS | 6.47 (5.68-7.37) | 3.46 (1.86-6.44) | 2.48 | 0.71 | 0.15 |
|  | Loss of Libido | FAERS | 6.38 (5.44-7.48) | 7.01 (4.22-11.64) | 2.41 | 1.94 | 0.34 |
| Renal and urinary disorders | Dysuria | FAERS | 3.72 (3.36-4.11) | 2.06 (1.26-3.37) | 1.73 | 0.2 | <0.01 |
|  | Pollakiuria | FAERS | 3.10 (2.79-3.44) | 1.88 (1.15-3.07) | 1.46 | 0.07 | 0.08 |
| Respiratory, thoracic and mediastinal disorders | **Interstitial lung disease** | **JADER** | **3.18 (2.81-3.59)** | **4.25 (3.42-5.29)** | **1.47** | **1.74** | **0.009** |
|  | **Interstitial lung disease** | **FAERS** | **1.42 (1.22-1.64)** | **8.75 (7.05-10.86)** | **0.26** | **2.77** | **<0.01** |
|  | Lung disorder | JADER | 0.55 (0.18-1.71) | 6.01 (3.31-10.91) | -2.93 | 1.56 | 0.000 |
| Skin and subcutaneous tissue disorders | Night Sweats | FAERS | 9.01 (8.38-9.69) | 2.99 (1.90-4.68) | 3.05 | 0.81 | <0.01 |
|  | Hyperhidrosis | FAERS | 2.04 (1.90-2.19) | 2.26 (1.75-2.91) | 0.91 | 0.75 | 0.44 |
| Vascular disorders | Hot Flush | FAERS | 41.47 (40.48-42.49) | 16.30 (14.32-18.57) | 5.34 | 3.81 | <0.01 |
|  | Deep vein thrombosis | JADER | 1.99 (1.20-3.31) | 0.66 (0.09-4.73) | 0.12 | -4.37 | 0.337 |

Red font showed the negative signals (aROR_025_<1 or IC_025_≤0);

P<0.05, showed the significant difference by Pearson χ^2^ test.

FAERS: FDA Adverse Event Reporting System; JADER: Japanese Adverse Drug Event Report; SOC: system organ class; PT: preferred term; IC_025_: adjusted the lower limit of 95% confidence interval of the information component of BCPNN; BCPNN: Bayesian confidence propagation neural network.
